# Synergistic oligodeoxynucleotide strongly promotes CpG-induced interleukin-6 production

**DOI:** 10.1186/s12865-017-0227-7

**Published:** 2017-10-04

**Authors:** Shireen Nigar, Yoshinari Yamamoto, Takuma Okajima, Suguru Shigemori, Takashi Sato, Tasuku Ogita, Takeshi Shimosato

**Affiliations:** 1 Interdisciplinary Graduate School of Science and Technology, Shinshu University, 8304 Minamiminowa, Kamiina, Nagano, 399-4598 Japan; 2 Department of Nutrition and Food Technology, Jessore University of Science and Technology, Jessore, Bangladesh; 3 Research Fellow of the Japan Society for the Promotion of Science (JSPS), 5-3-1 Kojimachi, Chiyoda-ku, Tokyo, 102-0083 Japan; 4 Graduate School of Science and Technology, Shinshu University, 8304 Minamiminowa, Kamiina, Nagano, 399-4598 Japan; 5 Department of Intestinal Ecosystem Regulation, Faculty of Medicine, University of Tsukuba, 1-1-1, Tennodai, Tsukuba, Ibaraki 3058575 Japan; 6 Metabologenomics Core, Transborder Medical Research Center, University of Tsukuba, 1-1-1, Tennodai, Tsukuba, Ibaraki 3058575 Japan; 7 Department of Pulmonology, Graduate School of Medicine, Yokohama City University, 3-9 Fukuura Kanazawa, Yokohama, 236-0004 Japan; 8 Institute for Biomedical Sciences, Shinshu University, 8304 Minamiminowa, Kamiina, Nagano, 399-4598 Japan; 9 Research Center for Fungal and Microbial Dynamism, Shinshu University, 8304 Minamiminowa, Kamiina, Nagano, 399-4598 Japan

**Keywords:** LGG, CpG-ODN, iSN-ODN, IL-6, Synergistic effect

## Abstract

**Background:**

Bacterial genomes span a significant portion of diversity, reflecting their adaptation strategies; these strategies include nucleotide usage biases that affect chromosome configuration. Here, we explore an immuno-synergistic oligodeoxynucleotide (iSN-ODN, named iSN34), derived from *Lactobacillus rhamnosus* GG (LGG) genomic sequences, that exhibits a synergistic effect on immune response to CpG-induced immune activation.

**Methods:**

The sequence of iSN34 was designed based on the genomic sequences of LGG. Pathogen-free mice were purchased from Japan SLC and maintained under temperature- and light-controlled conditions. We tested the effects of iSN34 exposure in vitro and in vivo by assessing effects on mRNA expression, protein levels, and cell type in murine splenocytes.

**Results:**

We demonstrate that iSN34 has a significant stimulatory effect when administered in combination with CpG ODN, yielding enhanced interleukin (IL)-6 expression and production. IL-6 is a pleotropic cytokine that has been shown to prevent epithelial apoptosis during prolonged inflammation.

**Conclusions:**

Our results are the first report of a bacterial-DNA-derived ODN that exhibits immune synergistic activity. The potent over-expression of IL-6 in response to treatment with the combination of CpG ODN and iSN34 suggests a new approach to immune therapy*.* This finding may lead to novel clinical strategies for the prevention or treatment of dysfunctions of the innate and adaptive immune systems.

**Electronic supplementary material:**

The online version of this article (10.1186/s12865-017-0227-7) contains supplementary material, which is available to authorized users.

## Background

Nucleic acids have been shown to be particularly potent molecular triggers of the innate immune response, not only providing a quick response against pathogens but also playing a role in shaping the adaptive immune response. These nucleic acids include CpG oligodeoxynucleotides (CpG ODNs) that are responsible for the immune stimulatory effect of bacterial DNA [1]. Several studies have demonstrated that bacterial DNA, as well as synthetic CpG ODNs (ODNs containing unmethylated CG dinucleotides and a phosphorothioate or chimeric backbone that renders these molecules nuclease resistant), have potent immunostimulatory effects [2]. In the present study, we investigated the immune response using ODN candidates from the *Lactobacillus rhamnosus* GG (LGG) genome. Notably, the genomes of diverse bacteria share DNA motifs that are rarely found in higher vertebrates [3–5]. These motifs include non-methylated CG dinucleotides that trigger cells expressing Toll-like receptor 9 (TLR9); this trigger results in the activation of natural killer (NK cells), B cells, monocytes, macrophages, and dendritic cells [2, 6–9], yielding an innate immune response characterized by the production of Th-1 cells and proinflammatory cytokines [10–12].

It is speculated that some intestinal bacteria exert beneficial effects, whereas others demonstrate deleterious effects. LGG chromosomal DNA has been shown to be a potent inducer of splenic B cell proliferation, CD86/CD69 expression, and cytokine production in mouse [13] and an efficient suppressor of allergic activity [14]. In addition, various bacterial strains (including *Lactobacillus, Lactococcus, Bifidobacterium, Enterobacter cloacae, Bacteroides fragilis*, *Enterococcus faecalis*, and *Escherichia coli*) have been shown to induce pro inflammatory mediators such as tumor necrosis factor (TNF) -α, interleukin (IL) -6, IL-12p70, and IL-23; these mediators may in turn be responsible for the induction and maintenance of chronic inflammatory responses [15–18]. One component shared among these inducing organisms is the nature of bacterial DNA. Bacterial DNA constitutes a pathogen-associated molecular pattern (PAMP) that is recognized by the vertebrate immune system, leading in turn to coordinated immune responses comprising both innate and acquired immunity [19]. Unmethylated CpG dinucleotides, which are present at high frequency in prokaryotic DNA but are rare in eukaryotic DNA [20, 21], are considered one such PAMP. Other research has suggested that bacterial DNA, alone or in combination with other bacterial products, triggers the release of IL-6, IL-12, interferon (IFN) -γ, and immunoglobulin (Ig) M [22]. In this context, the ability of bacterial DNA to induce IL-6 is of special interest. In fact, overproduction of IL-6 in vivo has been shown to cause various clinical symptoms and abnormalities (e.g., spiking fever, skin rash, arthritis, pericarditis, hepatosplenomegaly, and growth retardation) in laboratory models, which may explain the manifestations observed in patients with various inflammatory diseases, including rheumatoid arthritis (RA) and systematic-onset juvenile idiopathic arthritis (soJIA) [23, 24]. Here, we show that stimulation of mouse splenocytes with the combination of CpG-ODN and an immuno-synergistic ODN (iSN-ODN, which we have designated iSN34) derived from LGG genomic sequences yields significant up-regulation of IL-6 expression. We further demonstrate that this combination treatment leads to increased IL-6 expression in vivo, and that this increased expression enhances clinical symptoms and abnormalities. Indeed, CpG-motif-containing ODNs are widely studied as promising adjuvants for vaccines against a range of diseases, including infections, cancer (including kidney, skin, breast, uterine and immune malignancies [25, 26]), and allergies. Thus, our results are consistent with those previous studies; our observation of selective enhancement of IL-6 expression suggests that synergistic ODNs might be useful as vaccine adjuvants.

## Methods

### Animals

Pathogen-free female C57BL/6 mice (4 weeks of age) were purchased from Japan SLC (Shizuoka, Japan) and maintained under temperature- and light-controlled conditions. Mice were provided with ad libitum access to a standard diet of Labo MR Breeder (Nihon Nosan Co., Kanagawa, Japan) and sterile water. Mice were 6 to 7 weeks of age at the start of the study.

### ODNs

Endotoxin-free desalted phosphorothioated (PS) ODNs were synthesized by Integrated DNA Technologies, Inc. (Coralville, IA, USA). Each of the PS-ODNs was reconstituted in endotoxin-free water and passed through a 0.22-μm pore microfilter (Nihon Millipore K.K., Tokyo, Japan) prior to use. Mouse splenocytes were treated with equimolar amounts of CpG ODN 1555 [27], control ODN 1612 [28], CpG ODN 1585 [29], CpG ODN 2395 [30], or MsST (defined below) [31] (Table 1).

**Table 1:** ODN sequences

Name	5’-sequence-3’	Ref.
Ctr(ODN1612)	G^a^C^a^T^a^A^a^G^a^A^a^G^a^C^a^T^a^T^a^A^a^G^a^G^a^C^a^T	[28]
CpG-A(1585)	G^a^GGGTCAACGTTGAG^a^G^a^G^a^G^a^G^a^G	[29]
CpG-B(1555)	G^a^C^a^T^a^A^a^G^a^A^a^C^a^G^a^T^a^T^a^A^a^G^a^C^a^G^a^T	[27]
CpG-B(MsST)	C^a^A^a^G^a^G^a^A^a^C^a^G^a^T^a^T^a^G^a^T^a^A^a^T^a^C^a^A^a^C^a^T^a^G^a^A^a^A	[31]
CpG-C(2395)	T^a^C^a^G^a^T^a^C^a^G^a^T^a^T^a^T^a^T^a^C^a^G^a^G^a^C^a^G^a^C^a^G^a^C^a^ G^a^C^a^C^a^G	[30]
iSN34	T^a^T^a^C^a^C^a^T^a^A^a^A^a^G^a^C^a^T^a^T^a^G^a^A^a^G^a^G^a^C^a^C^a^T	This study

^a^Phosphorothioate bond

### Cells and cell culture

Splenocytes were prepared using standard methods [32, 33]. and were then cultured in triplicate or quadruplicate wells of a 24-well plate (Nalge Nunc International K.K., Tokyo, Japan) at 2 × 10^6^ cells/well in volumes of 1 mL/well of RPMI 1640 medium (Sigma-Aldrich) supplemented with 10% fetal calf serum (Sigma-Aldrich), 100 U/mL penicillin, 100 μg/mL streptomycin, 25 mM HEPES, 1.0 mM sodium pyruvate, nonessential amino acids, and 0.0035% 2-mercaptoethanol (2-ME).

### qPCR analysis

For analysis of gene expression, total RNA was isolated from ODN-stimulated mouse splenocytes and treated with DNase I (Macherey-Nagel GmbH & Co., Duren, Germany) for 15 min at room temperature; the nuclease was then heat-inactivated by incubation of the mixtures at 70 °C for 15 min [29, 34–36]. Thereafter, cDNA was prepared by reverse transcription from 1 μg of total RNA per sample using the prime Script® RT reagent kit (TaKaRa Bio, Inc., Tokyo, Japan). Equal volumes of cDNA were used for quantification of various cytokine cDNAs via real-time quantitative PCR (qPCR) using a Thermal Cycler Dice® Real Time System (TaKaRa Bio, Inc., Tokyo, Japan). The qPCR analyses were performed with SYBR Premix Ex Taq (TaKaRa Bio) using gene-specific primers, as described previously [32]. Primers for the β-actin- and IL-6-encoding genes were purchased from TaKaRa Bio. As a control, poly (A)^+^ RNA samples were used as templates to check for the presence of contaminating genomic DNA. Each pair of gene-specific primers included one primer designed to span an exon-intron junction, and the other designed to span the actual exon-intron boundary. The sensitivity of the reaction and amplification of contaminating products, such as self-annealed primers, were evaluated by amplifying serial dilutions of cDNA. For cross-sample comparison of results obtained following various treatments, levels of cytokine-encoding mRNA were first normalized to those of β-actin-encoding mRNA. Data are shown as the mean + standard deviation (SD) of one experiment representative of three independent experiments that yielded similar results.

### Wash-out assay

Splenocytes were prepared using standard methods [35]. Cells were cultured in a 24-well plate (Nalge Nunc International K.K., Tokyo, Japan) at 1 × 10^7^ cells/well in volumes of 1 mL/well of complete RPMI 1640 medium (Sigma-Aldrich) supplemented with 10% fetal calf serum, 100 U/mL penicillin, 100 μg/mL streptomycin, 25 mM HEPES, 1.0 mM sodium pyruvate, nonessential amino acids, and 0.0035% 2-ME. Cells were incubated in the presence of 3 μM iSN34 for 24 h at 37 °C in a 5% CO_2_ environment. Cells were then washed with fresh medium to remove the iSN34. Cells were resuspended in fresh medium supplemented with 3 μM CpG-B (ODN 1555) for 6 h and cytokine expression was detected by qPCR as described above. Data are shown as the mean + SD of one experiment representative of three independent experiments that yielded similar results.

### Cytokine quantification

IL-6 levels in cell culture supernatants after 48 h of various treatments were quantified using a commercially available ELISA kit (eBioscience Inc., San Diego, CA, USA) according to the manufacturer’s instructions.

### Intracellular staining

Splenocytes (2 × 10^6^ cells/well) were pre-incubated for 3 h in medium supplemented with either 0.625 μM iSN34 or an equivalent volume of water. Cells then were washed with medium to remove the ODNs, resuspended in medium containing 3.0 μM CpG-B (ODN 1555), and incubated for 12 h. After stimulation, cells were cultured for 4 h at 37 °C in RPMI 1640 medium supplemented with 10% fetal bovine serum, 100 U/mL of penicillin, 100 μg/mL of streptomycin, 10 μg/mL of brefeldin A, 2 μg/mL of ionomycin, and 20 ng/mL of phorbol 12-myristate 13-acetate. For intracellular staining, cells were fixed in 4% PFA for 15 min at room temperature, washed, and permeabilized by incubation for 15 min on ice; cells were then further incubated with phycoerythrin-labeled anti-mouse IL-6 antibody (Biolegend). Splenocytes were first stained with anti-mouse IL-6 antibody for 60 min on ice. Cells were washed and the percentages of CD19^+^ IL-6^+^ cells were determined using FACS Calibur (BD Biosciences). Data were acquired and analyzed using Flow Jo software. All analyses were carried out at least in triplicate; representative results are presented.

### In vivo study

In vivo experiments employed 4-week-old C57BL/6 female mice obtained and maintained as described above. After a preliminary acclimatization period of 2 weeks, mice (6 weeks of age) were sensitized by a total of 3 intraperitoneal (i.p.) injections (administered once every other week) of 200 μL of phosphate-buffered saline (PBS) + PBS, PBS (100 μL) + CpG-B (100 μg), iSN34 (20 μg) + PBS, or iSN34 (20 μg) + CpG-B (100 μg). For this study, MsST was used as the CpG-B. MsST is a strong immunostimulatory CpG ODN that is derived from the *lacZ* gene of *Streptococcus thermophilus* ATCC19258 and has an ability similar to that of the murine prototype CpG ODN (ODN 1555) to induce inflammatory cytokine production and cell proliferation [31]. Mice were subjected to euthanasia and necropsy at 1 week after the final i.p injection (i.e., at 11 weeks of age). At sacrifice, we measured body weight, spleen weight, and spleen length, and collected blood for further analysis.

### Statistical analysis

All statistical analyses were performed using a statistical software package (Prism 7, GraphPad, Inc., La Jolla, CA, USA). Two-tailed One-way ANOVA with a post-hoc Tukey-Kramer test was used to determine the significance of the differences in all experiments except for the body weight trends. Body weight gain changes were analyzed by Two-way ANOVA with post-hoc Bonferroni to compare treatment and time effects. Differences were considered significant at *p* < 0.05. Values for the in vivo experiment (spleen weight and spleen length) are presented using box and whisker plots. Other in vitro values are presented as means + SDs of three independent experiments (*n* = 9).

## Results

### Immune synergistic activity of LGG ODN and expression of *IL-6* mRNA by murine immune cells

**Fig. 1:**
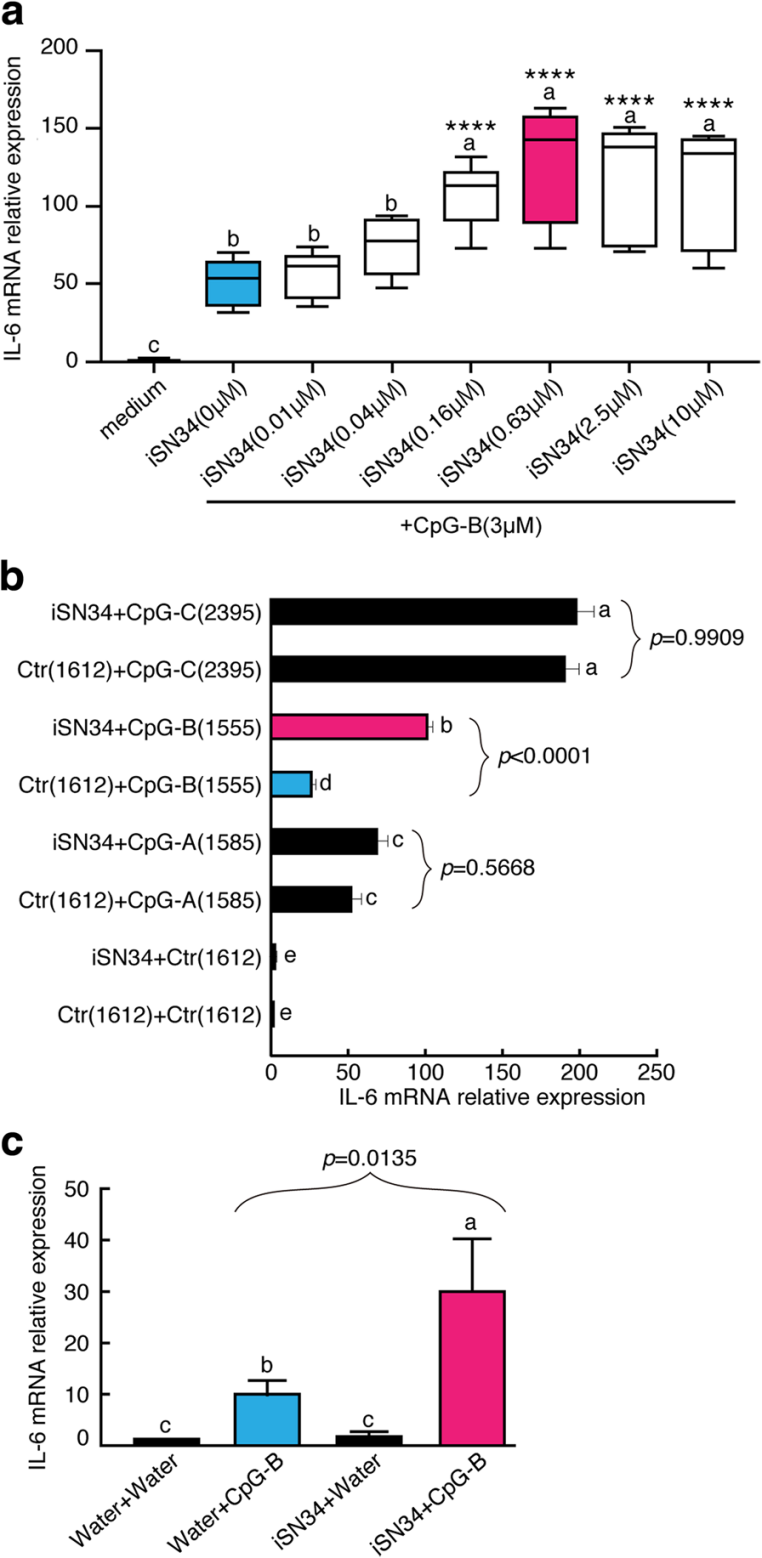
iSN34 was used to determine the optimal concentration of ODNs. **a** Mouse splenocytes were pre-incubated in medium for 3 h prior to exposure to iSN34 (at 0.01, 0.04, 0.16, 0.63, 2.5, or 10 μM) + CpG-B (ODN1555; at equimolar levels) or to ODN 1612 (control) for 6 h. Accumulation of *IL-6* mRNA was determined by qPCR. Results are shown as the ratio of *IL-6* mRNA levels for stimulated (iSN + CpG-B) versus ODN 1612-treated cells. **b** The synergistic effects of iSN34 (0.63 μM) were assessed in combination with CpG-A (ODN 1585), CpG-B (ODN 1555), CpG-C (ODN 2395), and Ctr 1612 (Control ODN). **c** Mouse splenocytes (1 × 10^7^ cells/mL) were pre-incubated in medium for 3 h prior to exposure to 3 μM ODN 1612 or iSN34 for 24 h. Cells then were washed with medium (to remove the ODNs) and resuspended in medium with 3 μM CpG-B (ODN 1555) for 6 h. Results are shown as *IL-6* mRNA expression (normalized to β-actin-encoding mRNA; see qPCR method) in stimulated cells in the wash-out assay. All assays were carried out at least three independent times in triplicate. Similar results were obtained from at least three different mice. Values are presented as mean + SD of three independent experiments, each performed in triplicate (*n* = 9). Values with different letters (i.e.*,* a, b, c, d, and e) were significantly different. ****p < 0.0001 vs. iSN34 (0 μM)

In this study, we analyzed the synergistic effects of ODNs that had been designed based on LGG DNA sequences. Candidates that were positive for synergistic effects in a preliminary screen were subjected to confirmation of synergistic activity by assessing the stimulation of *IL-6* mRNA expression in splenocytes (Additional file 1 Figure S1). In order to determine the effect of iSN34 on the expression and secretion of cytokines, we assessed *IL-6* transcription in mouse splenocytes exposed to the combination of iSN34 + CpG-B. To determine the optimal concentration of iSN34 for this assay, we examined *IL-6* mRNA accumulation in the presence of iSN34 at concentrations ranging from 0.01 to 10 μM. As shown in Fig. 1a, iSN34 (in the presence of CpG-B) exhibited dose-dependent stimulation of *IL-6* expression at iSN34 concentrations of 0.01 to 2.5 μM and the highest concentration of iSN34 determined at 0.63 μM. Next, we compared the activity of iSN34 (0.63 μM) when combined with CpG-A (ODN 1585), CpG-B (ODN 1555), CpG-C (ODN 2395), or negative control (ODN 1612) ODN. As shown in Fig. 1b, iSN34 exhibited synergy in the induction of *IL-6* mRNA expression when combined with A-type, B-type, or C-type CpG ODNs, while the combination of iSN34 and C-type CpG ODN did not yield significant induction of *IL-6* mRNA expression. These results suggested that synergy was strongest for the combination of iSN34 and CpG-B (ODN 1555). To further explore the activity of iSN34, we also examined the molecule’s effect on murine splenocytes in a wash-out assay. The culture was exposed to 3 μM iSN34 + CpG-B or control (water) for 24 h to monitor the immune synergistic activity after iSN34 removal. Interestingly, as shown in Fig. 1c, immune synergistic activity was maintained in splenocytes after iSN34 was removed from the culture, such that *IL-6* mRNA levels were effectively unchanged after wash-out of the ODNs.

### Induction of IL-6 production by exposure to low-concentration iSN34

**Fig. 2:**
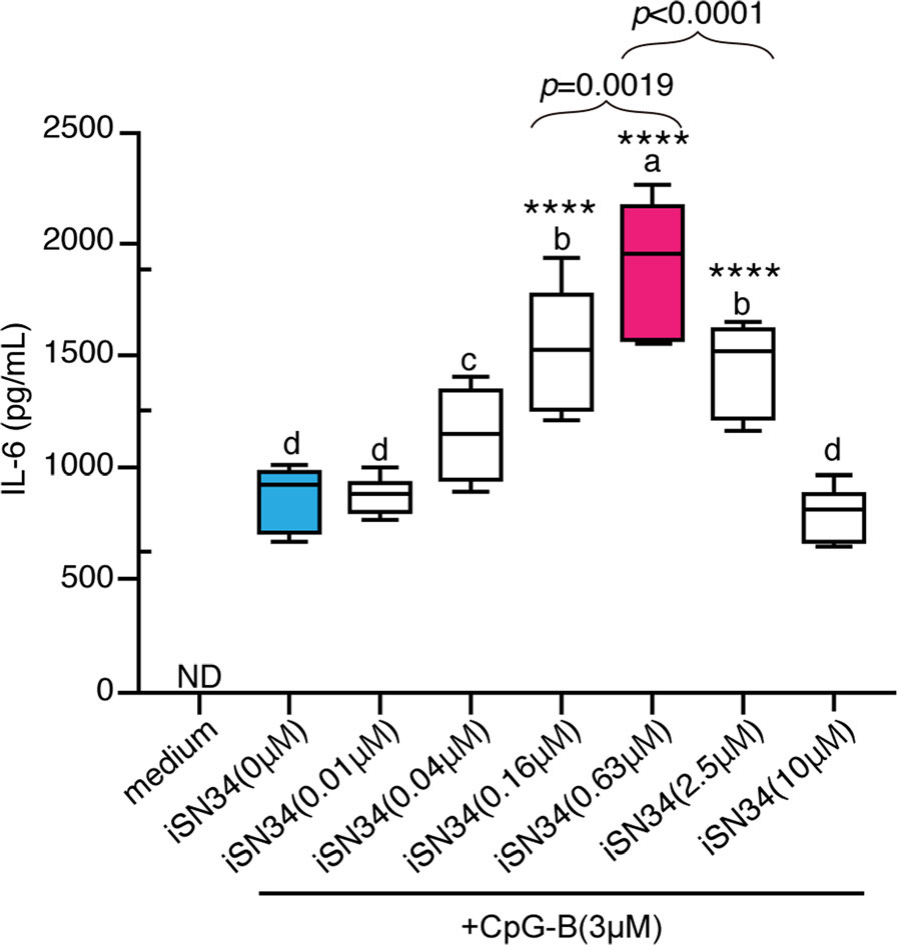
iSN34 with CpG-B enhances IL-6 production. Supernatants from stimulated cells were collected and IL-6 protein levels were measured by ELISA. Mouse splenocytes were harvested 48 h later and intracellular IL-6 protein levels were determined by ELISA. All assays were carried out at least three independent times in triplicate. Similar results were obtained from at least three different mice. Values are presented as mean + SD of three independent experiments, each performed in triplicate (*n* = 9). Values with different letters (i.e., a, b, c, d, e, and f) were significantly different. ****p < 0.0001 vs. iSN34(0 μM). ND; not detectable

We next investigated the secretion of IL-6 by murine splenocytes grown for 48 h in the presence of different concentrations of iSN34 (0.01 to 10 μM) + CpG-B; in this experiment, IL-6 levels in the spent medium were detected by ELISA. The results showed that the secretion of IL-6 was elevated in a dose-dependent fashion following iSN34 exposure, with peak IL-6 secretion detected in the presence of 0.63 μM iSN34 (Fig. 2). This result indicated that a low concentration of iSN34 can induce IL-6 production.

### Enhancement of the IL-6^+^ subpopulation in murine splenocytes

**Fig. 3:**
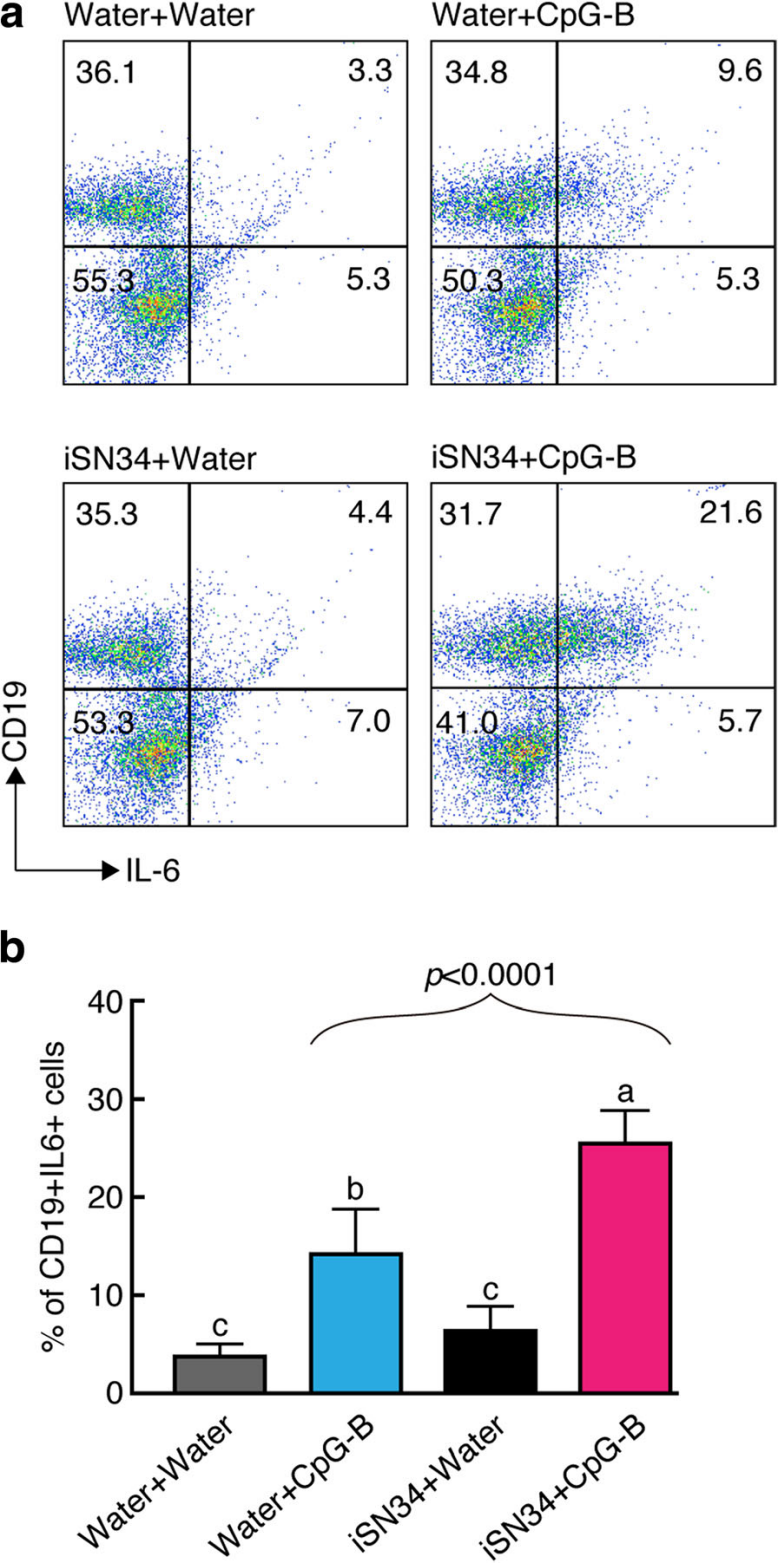
Representative flow cytometry plots. Dot plot of forward-angle versus right-angle light-scattering properties; the oval marks indicate the electronic windows used for analysis of fluorescence data for lymphocyte precursors; the rectangular boxes indicate the electronic windows used for analysis of fluorescence data for monocyte precursors with percentages. **a** A quadrant has been set to delineate the CD19 and IL-6 cells. Murine splenocytes were stimulated with water + water, water + CpG-B, iSN34 + water, or iSN34 + CpG-B for 12 h, and then sorted into CD19^+^ IL-6^+^ cells. **b** Mean percentage of IL-6^+^ CD19^+^ cells in the total population was determined in each group. Similar results were obtained from at least three different mice. Values are presented as mean + SD of three independent experiments, each performed in triplicate (*n* = 9). Values with different letters (i.e., a, b, c, and d) were significantly different (*p* < 0.01)

Next, we sought to investigate the effect of iSN34 exposure on a specific subpopulation of the splenocytes. Specifically, we used flow cytometry to classify the identity of IL-6^+^ mouse splenocyte cells following stimulation of the CpG-induced immune reaction by the synergistic ODN. We observed that the population of CD19^+^ IL-6^+^ cells was increased following induction with iSN34 + CpG-B (Fig. 3a). As shown in Fig. 3a, the percentage of CD19^+^ cells were significantly elevated in the cells treated with iSN34 + CpG-B. Additionally, this stimulation rendered the CD19^+^ IL-6^+^ subpopulation a significantly larger fraction of the total cell population (Fig. 3b). Thus, the synergistic effects of iSN34 + CpG-B reflected increased IL-6 expression in CD19^+^ B-lymophocytes.

### Effect of i.p. injection of iSN34 + CpG

**Fig. 4:**
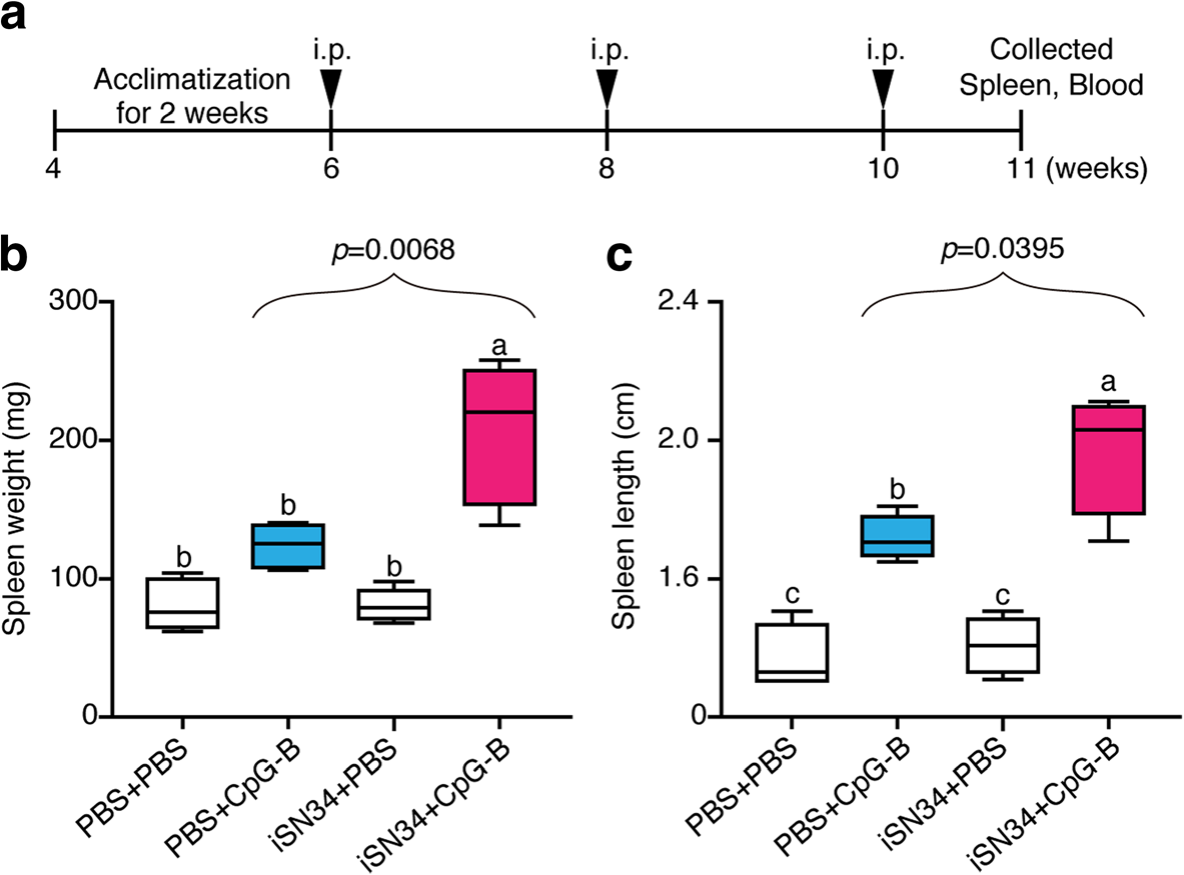
Cellular changes in spleens of mice following the in vivo trial. **a** Schematic design of the in vivo experiment, performed in female C57BL/6 mice. Six-week-old mice were sensitized by a total of three i.p. injections (administered once every other week) with 200 μL of PBS + PBS, PBS (100 μL) + CpG-B (100 μg), iSN34 (20 μg) + PBS, or iSN34 (20 μg) + CpG-B (100 μg). Mice were euthanized at 11 weeks of age. **b** Spleen weight (mg). **c** Spleen length (cm). Results in panels **b** and **c** are presented using box and whisker plots. The central bar in each box indicates the median, with boxes extending to the 25th and 75th percentile values; whiskers extend to the minimum and maximum values. Values with different letters (i.e., a, b, c, and d) were significantly different (*p* < 0.01)

We next extended our analysis of the synergistic effects of iSN34 + CpG by testing this treatment in vivo (Fig. 4a). All four groups of mice (*n* = 4/group; injected i.p. with PBS + PBS, PBS + CpG-B, iSN34 + PBS, or iSN34 + CpG-B) exhibited typical weight gain during the course of the study, and there was no significant difference among the 4 groups in terms of terminal body weight (Additional file 2 Figure S2). We did, however, note hypertrophy (splenomegaly) of the spleen in mice treated with iSN34 + CpG-B compared to the spleens of the other groups (Additional file 3 Figure S3); these effects were significant whether assessed as spleen weight (Fig. 4b) or length (Fig. 4c). Our findings suggest that the combined use of iSN34 and CpG-B may find application in modifying the innate immune response.

## Discussion

Our study revealed that iSN34 exhibits immune synergistic effects when combined with CpG-ODN. The activity of iSN34 was associated with multiple types of CpG-ODNs. We showed the synergistic activity of iSN34 + CpG at the level of *IL-6* mRNA accumulation, IL-6 protein secretion, and IL-6^+^ cell production following in vitro exposure of cultured splenocytes. Among the tested ODNs, only iSN34 exhibited these effects, which were observed only when combined with CpG ODN. Previous work has shown that CpG ODN is an extremely potent stimulator of dendritic cells and macrophages, causing the induction of Th-1-associated cytokines such as IL-6 and IL-12 [37]. In the present work, the ODN iSN34 was able (in combination with CpG) to stimulate the production of IL-6, suggesting that iSN34 would be a highly effective adjuvant for Th-1-mediated vaccines.

Thus, our results indicated that the combination of iSN34 + CpG strongly induces the expression of IL-6, and therefore might be useful for the prevention or treatment of diseases associated with inflammatory disorders, including RA, inflammatory bowel disease (IBD), multiple sclerosis, systematic-onset juvenile chronic arthritis (JCA), osteoporosis, and psoriasis [38–41]. However, other work has shown that IL-6 acts as a potent stimulator of B-cell proliferation [42], plasma cell survival [43], and antibody production [44], effects that might be at odds with the prevention or treatment of some diseases. Further work will be needed to address the combination of these distinct effects.

We observed that iSN34 exhibited greater synergy when combined with CpG-B (Fig. 1b). On the other hand, iSN34 did not display synergy in combination with CpG-C. These results indicated that CpG-B is the appropriate synergistic inducer for use with iSN34. Our studies also showed that co-administration of iSN34 + CpG ODN had synergistic effects in a wash-out assay. Specifically, we demonstrated that splenocytes pretreated with iSN34 maintain the ability to stimulate the immune response even after the iSN34 is washed out, suggesting that the immune synergistic effect of iSN34 may be mediated via stimulation of cell signaling. Assessment of the immune synergistic effect of iSN34 + CpG-B by measuring IL-6 protein secretion (via ELISA) demonstrated that 0.63 μM iSN34 yielded peak synergistic efficacy under our experimental conditions.

CD19 is one of the most reliable biomarkers for B cells that can receive positive stimuli from a variety of cytokines, including IFN, IL-4 and IL-6; such stimulation leads to proliferation, differentiation, cytokine production, and other effector functions [45]. In the present work, B cells exposed to the combination of CpG and iSN34 showed significant up-regulation of CD19 expression compared to controls. To clarify whether specific subpopulations of cells were selectively activated by iSN34, we further characterized IL-6producing splenocytes using flow cytometry. This flow cytometric analysis revealed that the proportion of IL-6secreting CD19^+^ B-cells in the spleen increased over two-fold in mice treated with iSN34 + CpG-B, compared to the levels seen in the control group (Fig. 3c). These findings could be relevant for the study of inflammation.

We also found that co-administration of iSN34 + CpG-B in vivo in healthy mice was sufficient to cause expansion of this cell type, yielding increased spleen weight and length. Notably, this treatment did not result in significant changes in terminal body weight compared to control groups, suggesting that iSN34 was not associated with gross toxicity (as might be implied by weight loss or attenuation of weight gain). We did, however, detect hypertrophy of the spleen in mice sensitized by i.p. injection with iSN34 + CpG-B. In previous work, we demonstrated that CpG-B is involved in activating the innate immune response and induces splenomegaly [29, 46]. Indeed, splenomegaly has been considered an important complication of acute and chronic disease for more than 100 years [47–49].

Notably, iSN34 does not provide significant synergistic induction of inflammatory cytokines (TNF-α, IFN-γ) other than IL-6 (data not shown). Given this observation, combined therapies incorporating iSN34 may significantly complement and further enhance the positive effects of mucosal immunity. In summary, our study demonstrated that iSN34 acts synergistically in combination with CpG ODN in the induction of IL-6. This multifunctional cytokine was originally identified as a T-cell-derived factor, and has subsequently been shown to induce the differentiation of activated B-cells into antibody-producing cells with biological activities that include the regulation of immune response, inflammation, and hematopoiesis [23]. This synergy may represent a new modality for the treatment of systematic inflammatory disorders.

## Conclusions

The new insights provided by this study suggest a new concept of synergistic ODN activity distinct from that described in previous ODN studies. However, the implications of this synergy for clinical symptoms or disease risk are unknown. These facts suggest that this immune synergy represents a wide-open research field with good scientific prospects, and could yield improvements in the prevention and treatment of immune disorders. However, further preclinical and clinical investigations will be needed to understand how specific targeting of the IL-6 pathway can be applied in disease treatment.

## Additional files

Additional file 1: Figure S1. Analysis of *IL-6* mRNA expression in mouse splenocytes, as assessed by qPCR. Mouse splenocytes (2 × 10^6^ cells/mL) were pre-incubated in medium for 3 h prior to exposure to 3 μM iSN candidates (No. 1–50), to CpG-B (ODN 1555), or to ODN 1612 (control) for 6 h. The results are presented as the mean + SD of at least three independent experiments, each performed in triplicate. **** *p* < 0.0001 vs. [Ctr (1612) + CpG-B] (blue). Red: [iSN34 + CpG-B], Orange: [iSN35 + CpG-B]. (TIFF 16522 kb)

Additional file 2: Figure S2. Body weight trends in female C57BL/6 mice (from 6 to 11 weeks of age) during treatment with iSN34 + CpG-B or control regimens. Body weights were measured once weekly. Data presented are the average weight per group. Data are presented as mean ± SE. (TIFF 2046 kb)

Additional file 3: Figure S3. Changes in spleen size in mice after administration of iSN34 + CpG-B or control regimens. The picture shows representative spleens from one animal of each of the four groups: PBS + PBS, PBS + CpG-B, iSN34 + PBS, and iSN34 + CpG-B. (TIFF 5405 kb)

## Abbreviations

CpG ODN: Cytosine-phosphate-guanine oligodeoxynucleotide
IFN: Interferon
IL: Interleukin
iSN ODN: Immuno-synergistic oligodeoxynucleotide
JCA: Systematic-onset juvenile chronic arthritis
LGG: *Lactobacillus rhamnosus* GG
PAMP: Pathogen-associated molecular pattern
PBS: Phosphate-buffered saline
RA: Rheumatoid arthritis
soJIA: Systematic-onset juvenile idiopathic arthritis
TNF: Tumor necrosis factor
