# Supplementary figures and images for: Synergistic oligodeoxynucleotide strongly promotes CpG-induced interleukin-6 production
Source: BMC Immunol. 2017 Oct 4;18:44. doi: 10.1186/s12865-017-0227-7 (PMC5628431; doi:10.1186/s12865-017-0227-7)

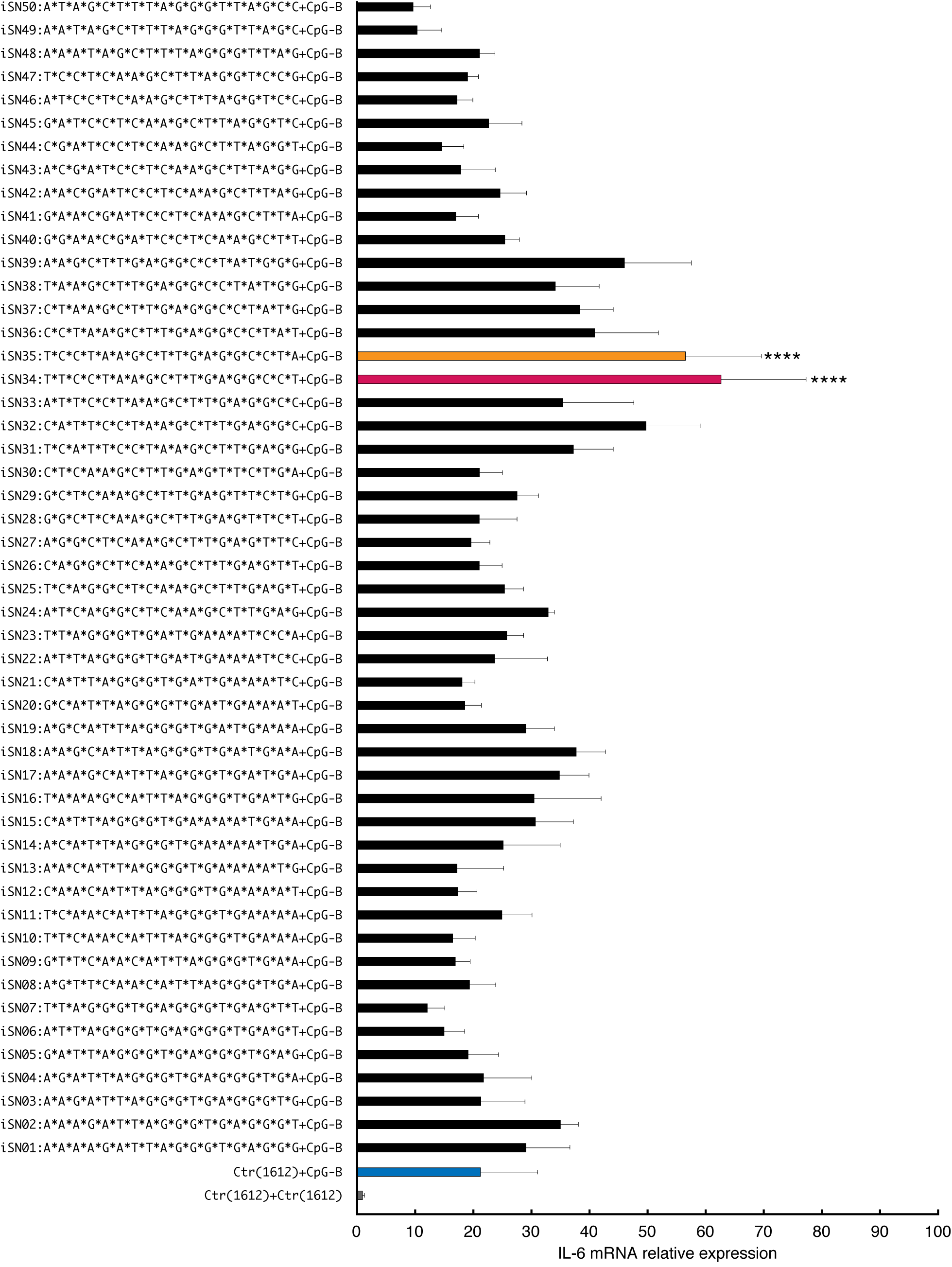

Supplement: Supplementary file 1 — Analysis of IL-6 mRNA expression in mouse splenocytes, as assessed by qPCR. Mouse splenocytes (2 × 10^6^ cells/mL) were pre-incubated in medium for 3 h prior to exposure to 3 μM iSN candidates (No. 1–50), to CpG-B (ODN 1555), or to ODN 1612 (control) for 6 h. The results are presented as the mean + SD of at least three independent experiments, each performed in triplicate. **** p < 0.0001 vs. [Ctr (1612) + CpG-B] (blue). Red: [iSN34 + CpG-B], Orange: [iSN35 + CpG-B]. (TIFF 16522 kb) [file 12865_2017_227_MOESM1_ESM.tif]

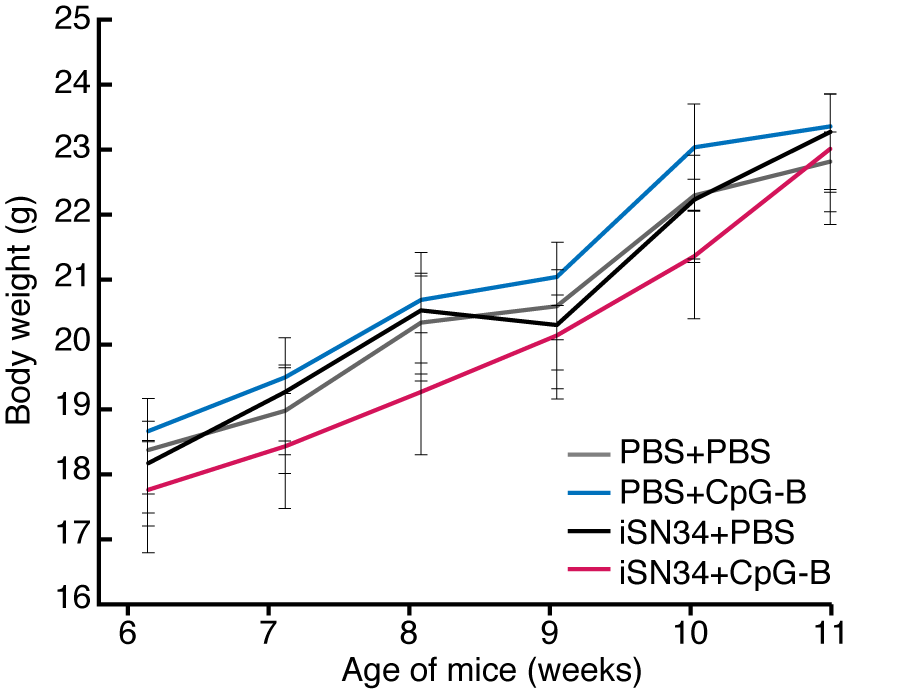

Supplement: Supplementary file 2 — Body weight trends in female C57BL/6 mice (from 6 to 11 weeks of age) during treatment with iSN34 + CpG-B or control regimens. Body weights were measured once weekly. Data presented are the average weight per group. Data are presented as mean ± SE. (TIFF 2046 kb) [file 12865_2017_227_MOESM2_ESM.tif]

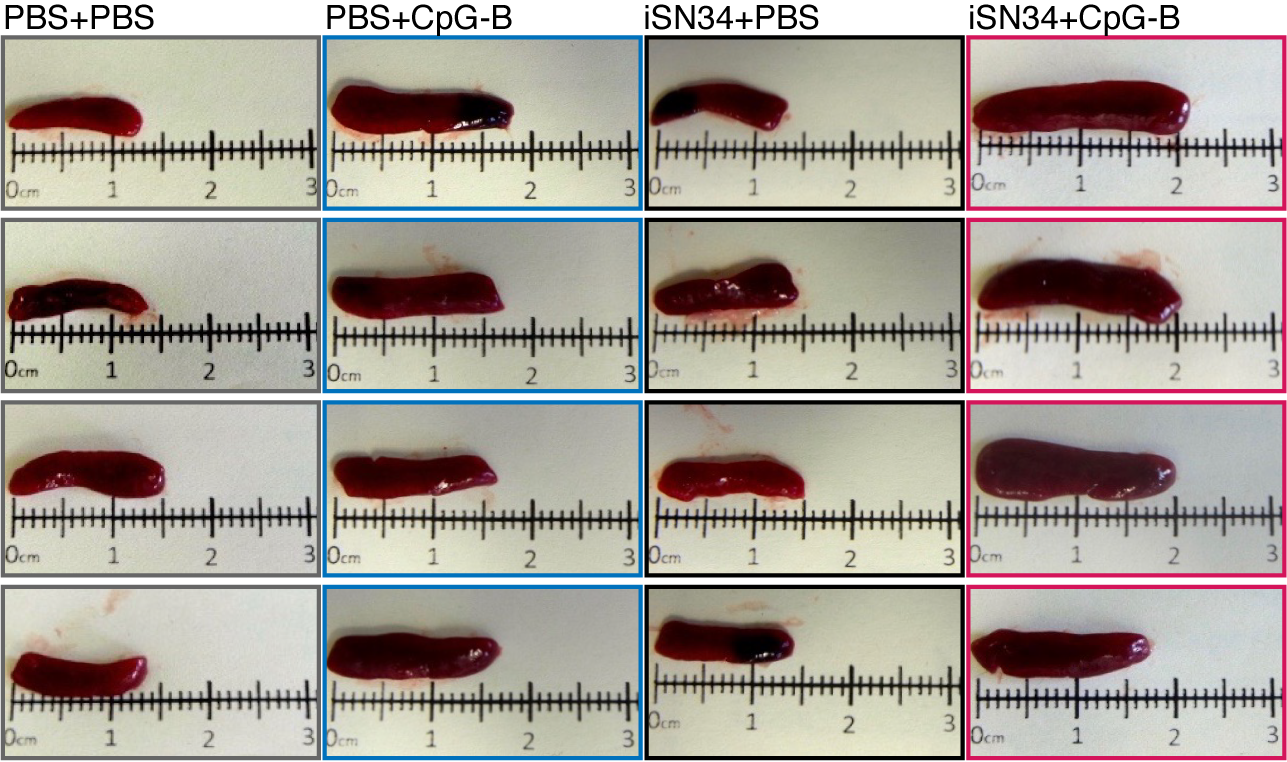

Supplement: Supplementary file 3 — Changes in spleen size in mice after administration of iSN34 + CpG-B or control regimens. The picture shows representative spleens from one animal of each of the four groups: PBS + PBS, PBS + CpG-B, iSN34 + PBS, and iSN34 + CpG-B. (TIFF 5405 kb) [file 12865_2017_227_MOESM3_ESM.tif]
